# Supplementary material for: Structural basis for activity switching in polymerases determining the fate of let-7 pre-miRNAs
Source: Nat Struct Mol Biol. 2024 Jul 25;31(9):1426–38. doi: 10.1038/s41594-024-01357-9 (PMC11402785; doi:10.1038/s41594-024-01357-9)
Supplement: Supplementary file 1 — List of RNA and DNA synthetic oligonucleotides used in cloning, in complex assembly or in functional assays. [file 41594_2024_1357_MOESM1_ESM.pdf]

nature structural & molecular biology

Supplementary information

https://doi.org/10.1038/s41594-024-01357-9

Structural basis for activity switching in polymerases determining the fate of let-7 pre-miRNAs

In the format provided by the authors and unedited

Supplementary Table 1: List of synthetic nucleotides

Name	Sequence (5' to 3')
TUT7_M1_Fw	5'-TACTTCCAATCCATG GGCGATACCGCCAAGC-3'
TUT7_S1495_Rv	5'-TATCCACCTTTACTGCT GCTCTCTTGCTGTGTCCGC-3'
TUT4_M1_Fw	5'-TACTTCCAATCCATG GAAGAGAGCAAGACCC-3'
TUT4_V153_Fw	5'-TACTTCCAATCCATG CCCAGCTCTCCTGCCGAGG-3'
TUT4_E254_Fw	5'-TACTTCCAATCCATG GAGATGGACTACCTGGAAAACGC-3'
TUT4_V1315_Rv	5'-TATCCACCTTTACTGCT CAGCAGGCTCTTTCTCTTGGG-3'
TUT4_E1644_Rv	5'-TATCCACCTTTACTGCT CTCGGACACATTGCCTCTA-3'
TUT4_R283A_R286A_Fw	5'-CAGGCCGAGGAAGCCCTGGAAGCCGACCACATCTTC-3'
TUT4_R283A_R286A_Rv	5'-CTTCAGTCCCAGTCTCTGCT-3'
TUT4_H320A_K321A_Fw	5'-ATCCAGGGCGCCGCCGCCCACATCAAAGAG-3'
TUT4_H320A_K321A_Rv	5'-CTTCAGTCCCAGTCTCTGCT-3'
TUT4_K324A_R327A_Fw	5'-CACAAGCACATCGCCGAGAAGGCCCACAAGAAGAAT-3'
TUT4_K324A_R327A_Rv	5'-GGCGCCCTGGATATTCTCGA-3'
TUT4_N688A_L691A_Fw	5'-GCCAGAAGCCTGGCCAGCCAGGCCGTGTACGAGTAC-3'
TUT4_N688A_L691A_Rv	5'-CACGTTCCGCTTCACGCTGA-3'
TUT4_K919A_K920A_Fw	5'-TGCTCCATCTGCGCCGCCGACGGCCACTCC-3'
TUT4_K919A_K920A_Rv	5'-CACGATGGTAGGTGGCTTGC-3'
TUT4_D009A_D1011A_Fw	5'-AACGATAGATGCGCCAGAGTGGCCGGAAAGATCGGC-3'
TUT4_D009A_D1011A_Rv	5'-TGGGGCCAGCTCGCCGTCTG-3'
Lin28A_M1_Fw	5'-TACTTCCAATCCATG GGCTCTGTGTCCAACC-3'
Lin28A_R209_Rv	5'-TATCCACCTTTACTGCT ATTCTGCGCCTCCGGCAGC-3'
Pre-let-7g	5'-UGAGGUAGUAGUUUGUACAGUUUGAGGGUCUAUGAUACCACCCGGUACAGGAGAUAACUGUACAGGCCACUGCCUUGC-3'
Pre-let-7g_3'CY	5'-3'CY-UGAGGUAGUAGUUUGUACAGUUUGAGGGUCUAUGAUACCACCCGGUACAGGAGAUAACUGUACAGGCCACUGCCUUGC-3'
Pre-let-7g_1U_3'CY	5'-3'CY-UGAGGUAGUAGUUUGUACAGUUUGAGGGUCUAUGAUACCACCCGGUACAGGAGAUAACUGUACAGGCCACUGCCUUGC-3'
