# Supplementary material for: Structural basis for activity switching in polymerases determining the fate of let-7 pre-miRNAs
Source: Nat Struct Mol Biol. 2024 Jul 25;31(9):1426–38. doi: 10.1038/s41594-024-01357-9 (PMC11402785; doi:10.1038/s41594-024-01357-9)
Supplement: Supplementary file 2 — Reporting Summary [file 41594_2024_1357_MOESM2_ESM.pdf]

nature portfolio

Corresponding author(s): Robert Gilbert

Last updated by author(s): Mar 29, 2024

Reporting Summary

Nature Portfolio wishes to improve the reproducibility of the work that we publish. This form provides structure for consistency and transparency in reporting. For further information on Nature Portfolio policies, see our Editorial Policies and the Editorial Policy Checklist.

Statistics

For all statistical analyses, confirm that the following items are present in the figure legend, table legend, main text, or Methods section.

n/a	Confirmed	
☐	✓	The exact sample size (*n*) for each experimental group/condition, given as a discrete number and unit of measurement
☒	☐	A statement on whether measurements were taken from distinct samples or whether the same sample was measured repeatedly
☒	☐	The statistical test(s) used AND whether they are one- or two-sided<br>*Only common tests should be described solely by name; describe more complex techniques in the Methods section.*
☒	☐	A description of all covariates tested
☒	☐	A description of any assumptions or corrections, such as tests of normality and adjustment for multiple comparisons
☐	✓	A full description of the statistical parameters including central tendency (e.g. means) or other basic estimates (e.g. regression coefficient) AND variation (e.g. standard deviation) or associated estimates of uncertainty (e.g. confidence intervals)
☒	☐	For null hypothesis testing, the test statistic (e.g. *F*, *t*, *r*) with confidence intervals, effect sizes, degrees of freedom and *P* value noted<br>*Give P values as exact values whenever suitable.*
☒	☐	For Bayesian analysis, information on the choice of priors and Markov chain Monte Carlo settings
☒	☐	For hierarchical and complex designs, identification of the appropriate level for tests and full reporting of outcomes
☒	☐	Estimates of effect sizes (e.g. Cohen's *d*, Pearson's *r*), indicating how they were calculated

Our web collection on statistics for biologists contains articles on many of the points above.

Software and code

Policy information about availability of computer code

Data collection	EPU Software version 2.10 was used for cryo-EM data collection.
Data analysis	CryoSPARC software version 4 was used for image analysis to determine 3D reconstructions, and DeepEMhancer version 2.0 for post-processing. Structures were built using Coot (version 0.9.8.8) and Phenix (version 1.19.2-4158), and structures were displayed using either Pymol (Version 2.0) or ChimeraX version 1.6.

For manuscripts utilizing custom algorithms or software that are central to the research but not yet described in published literature, software must be made available to editors and reviewers. We strongly encourage code deposition in a community repository (e.g. GitHub). See the Nature Portfolio guidelines for submitting code & software for further information.

Data

Policy information about availability of data

All manuscripts must include a data availability statement. This statement should provide the following information, where applicable:

- Accession codes, unique identifiers, or web links for publicly available datasets
- A description of any restrictions on data availability
- For clinical datasets or third party data, please ensure that the statement adheres to our policy

The density maps and atomic coordinates reported in this paper have been deposited in the EMDB and RCSB PDB, respectively, with accession codes as follows: TUT7 apo structure EMD-16825 and PDB ID 8OEF; TUT7 bound with pre-let-7g and UTPαS EMD-17084 and PDB ID 8OPP; TUT7 bound with pre-let-7g and LIN28A

conformation-I EMD-17086 and PDB ID 8OPS; TUT7 bound with pre-let-7g and LIN28A conformation-II EMD-17087 and PDB ID 8OPT; TUT4 bound with pre-let-7g and LIN28A (equivalent to TUT7 ternary conformation-I) EMD-17164 and PDB ID 8OST.

Human research participants

Policy information about studies involving human research participants and Sex and Gender in Research.

Reporting on sex and gender	N/A
Population characteristics	N/A
Recruitment	N/A
Ethics oversight	N/A

Note that full information on the approval of the study protocol must also be provided in the manuscript.

Field-specific reporting

Please select the one below that is the best fit for your research. If you are not sure, read the appropriate sections before making your selection.

☒ Life sciences ☐ Behavioural & social sciences ☐ Ecological, evolutionary & environmental sciences

For a reference copy of the document with all sections, see nature.com/documents/nr-reporting-summary-flat.pdf

Life sciences study design

All studies must disclose on these points even when the disclosure is negative.

Sample size	N/A
Data exclusions	During image analysis, image data which are of poor quality e.g. show movement or contamination, or which represent a minor conformational or compositional state are excluded, following the established CryoSPARC pipeline.
Replication	Assessment of resolution involves a "Gold-standard" Fourier shell correlation in which the data are separated into two halves before reconstruction, and resolution is assessed by the level of agreement between the maps. Thus replication of the structures is inherent to the analysis pipeline.
Randomization	Even-odd separation to two half datasets. This is standard and reckoned to be the best way to separate image data randomly.
Blinding	N/A

Reporting for specific materials, systems and methods

We require information from authors about some types of materials, experimental systems and methods used in many studies. Here, indicate whether each material, system or method listed is relevant to your study. If you are not sure if a list item applies to your research, read the appropriate section before selecting a response.

Materials & experimental systems

n/a	Involved in the study	
☒	☐	Antibodies
☒	☐	Eukaryotic cell lines
☒	☐	Palaeontology and archaeology
☒	☐	Animals and other organisms
☒	☐	Clinical data
☒	☐	Dual use research of concern

Methods

n/a	Involved in the study	
☒	☐	ChIP-seq
☒	☐	Flow cytometry
☒	☐	MRI-based neuroimaging
