# Supplementary material for: Structural basis for activity switching in polymerases determining the fate of let-7 pre-miRNAs
Source: Nat Struct Mol Biol. 2024 Jul 25;31(9):1426–38. doi: 10.1038/s41594-024-01357-9 (PMC11402785; doi:10.1038/s41594-024-01357-9)

Source Data for Figure 6A-C and E-H

6A:

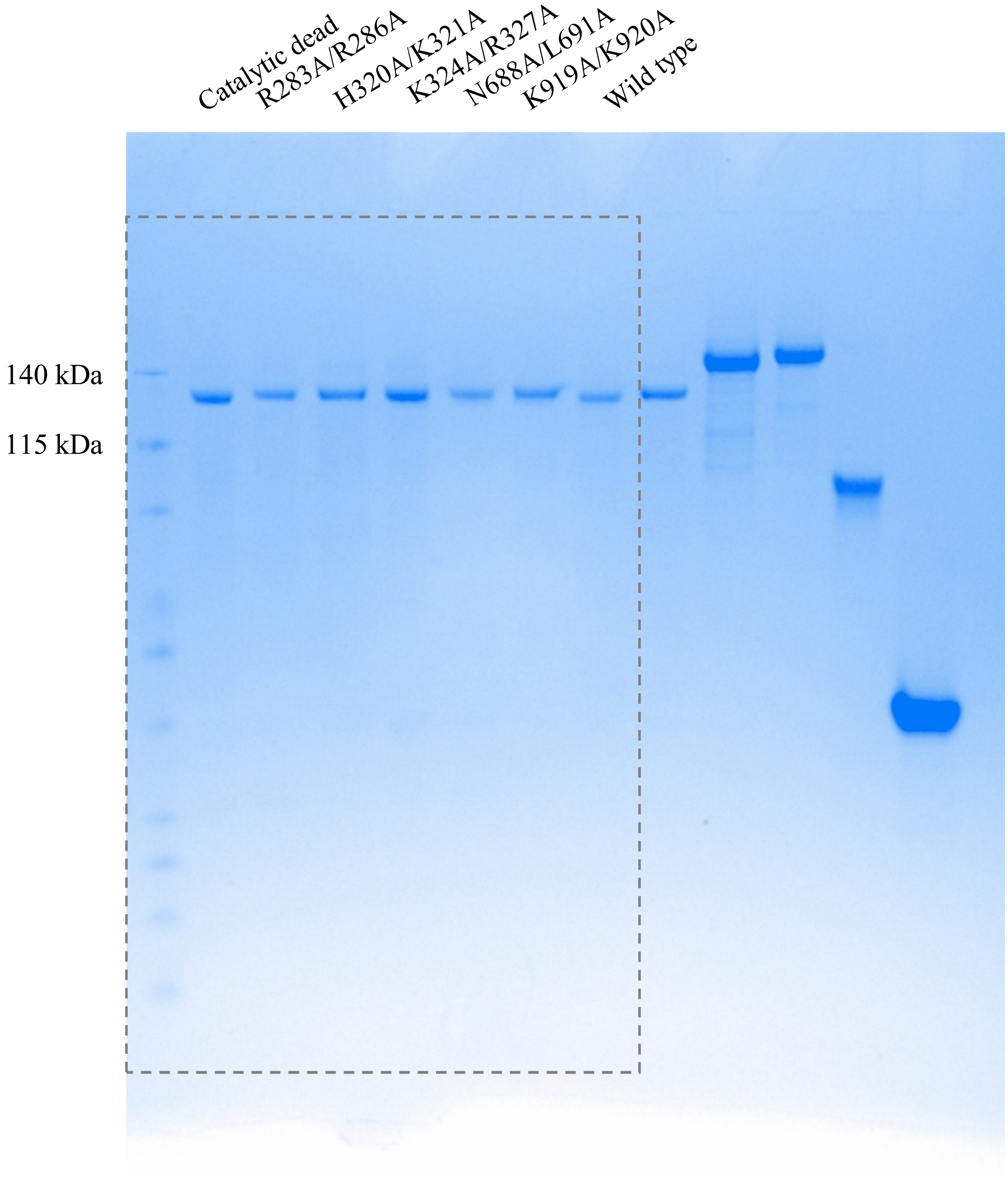

6B and 6C:

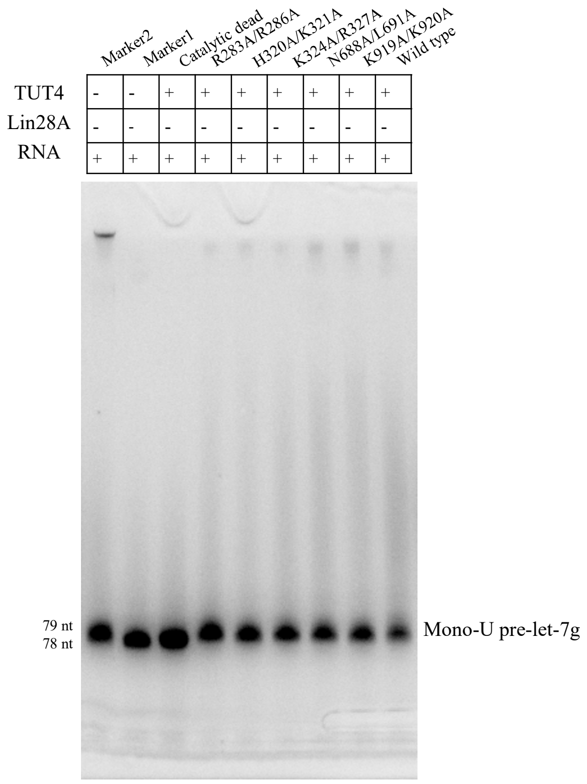
Marker2
Marker1
Catalytic dead
R283A/R286A
H320A/K321A
K324A/R327A
N688A/L691A
K919A/K920A
Wild type
TUT4 - - + + + + + + +
Lin28A - - - - - - - - -
RNA + + + + + + + + +
79 nt
78 nt
Mono-U pre-let-7g

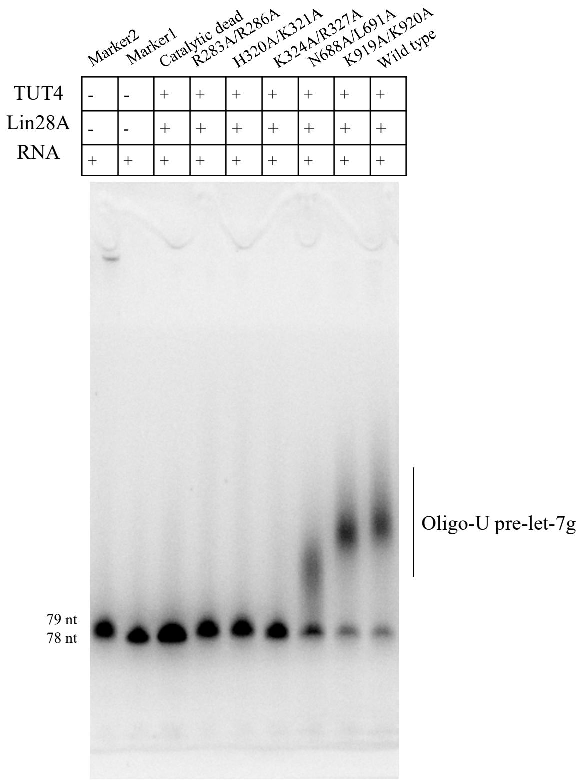
Marker2
Marker1
Catalytic dead
R283A/R286A
H320A/K321A
K324A/R327A
N688A/L691A
K919A/K920A
Wild type
TUT4 - - + + + + + + +
Lin28A - - + + + + + + +
RNA + + + + + + + + +
Oligo-U pre-let-7g
79 nt
78 nt

6E:

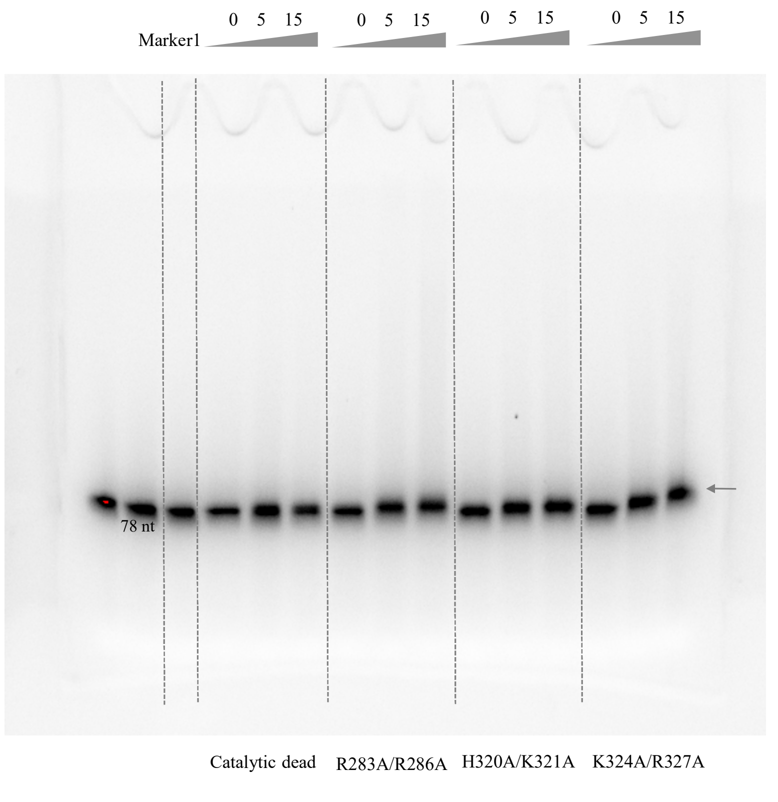
Marker1
0 5 15
0 5 15
0 5 15
0 5 15
78 nt
Mono-U pre-let-7g
Catalytic dead
R283A/R286A
H320A/K321A
K324A/R327A

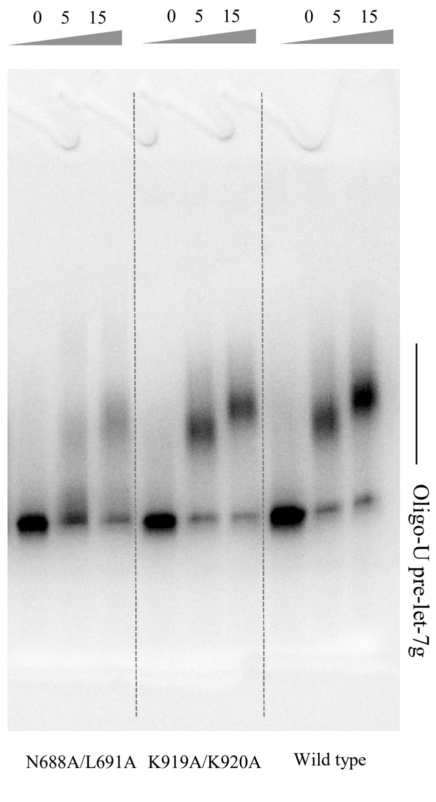
0 5 15
0 5 15
0 5 15
Oligo-U pre-let-7g
N688A/L691A
K919A/K920A
Wild type

6F and G:

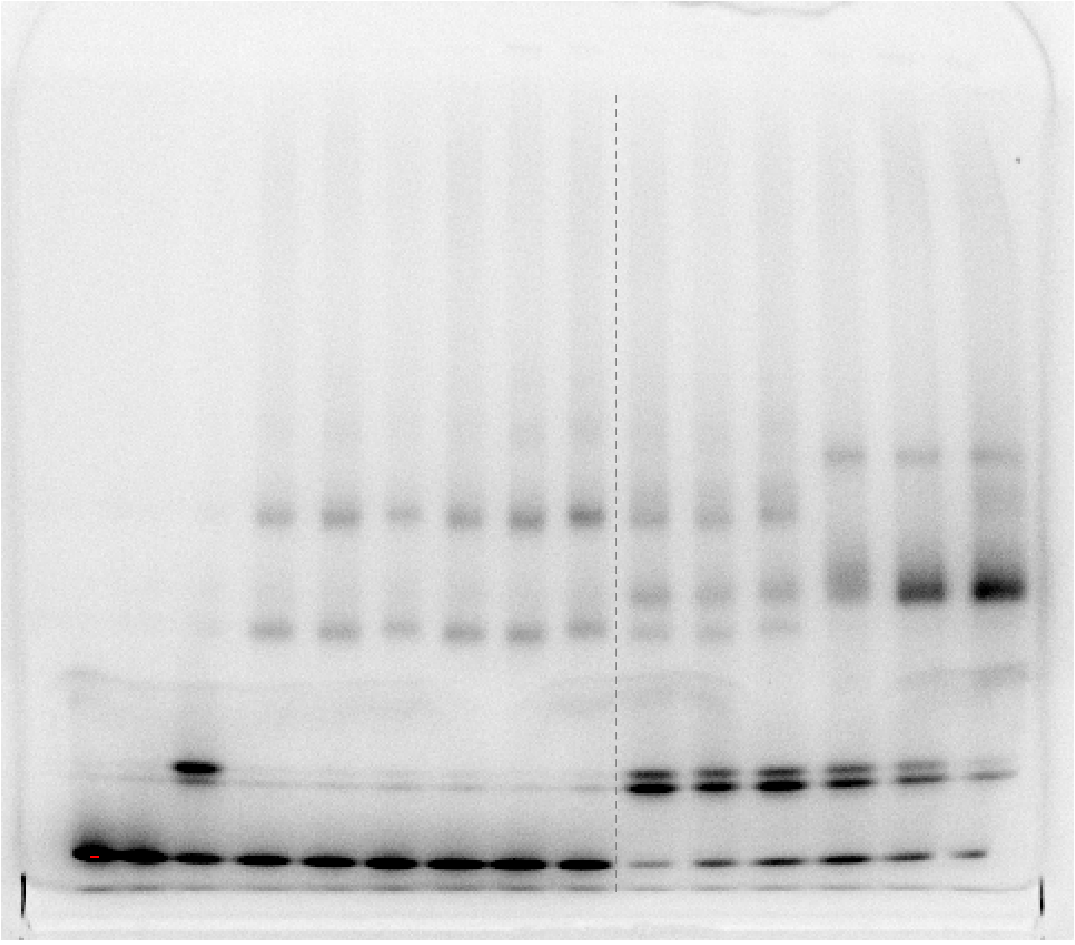

TUT4	-	-	+	+	+	+	+	+
Lin28A	-	+	-	-	-	-	-	-
RNA	+	+	+	+	+	+	+	+
			R283A/R286A	H320A/K321A	K324A/R327A	N688A/L691A	K919A/K920A	Wild type

TUT4	+	+	+	+	+	+
Lin28A	+	+	+	+	+	+
RNA	+	+	+	+	+	+
	R283A/R286A	H320A/K321A	K324A/R327A	N688A/L691A	K919A/K920A	Wild type

6H:

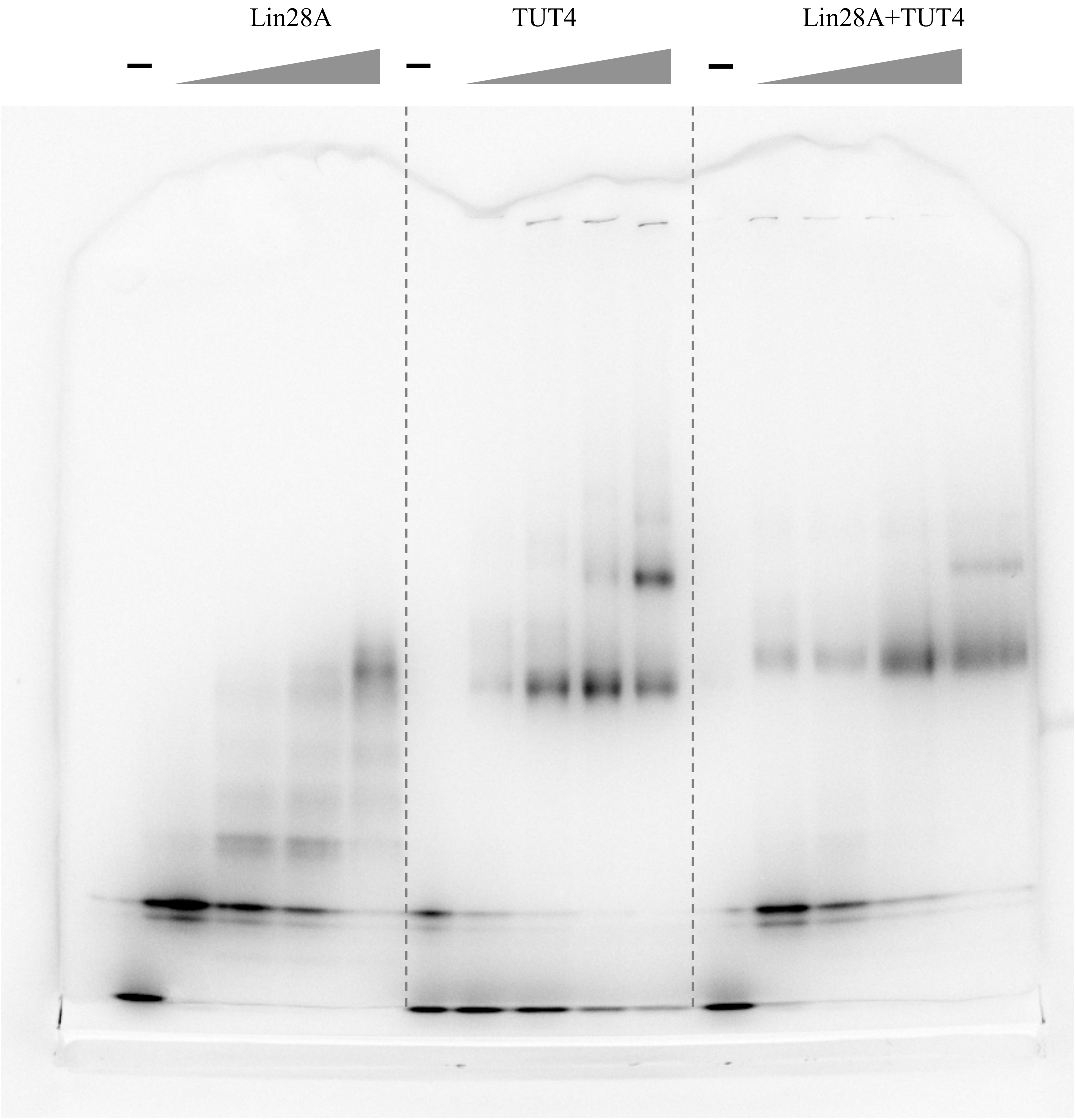

Supplement: Supplementary file 7 — Uncut gel images for Fig. 6 panels. [file 41594_2024_1357_MOESM7_ESM.pdf]
