# Supplementary figures and images for: Structural basis for activity switching in polymerases determining the fate of let-7 pre-miRNAs
Source: Nat Struct Mol Biol. 2024 Jul 25;31(9):1426–38. doi: 10.1038/s41594-024-01357-9 (PMC11402785; doi:10.1038/s41594-024-01357-9)

Source Data for Extended Data figure 1

ED figure 1B:

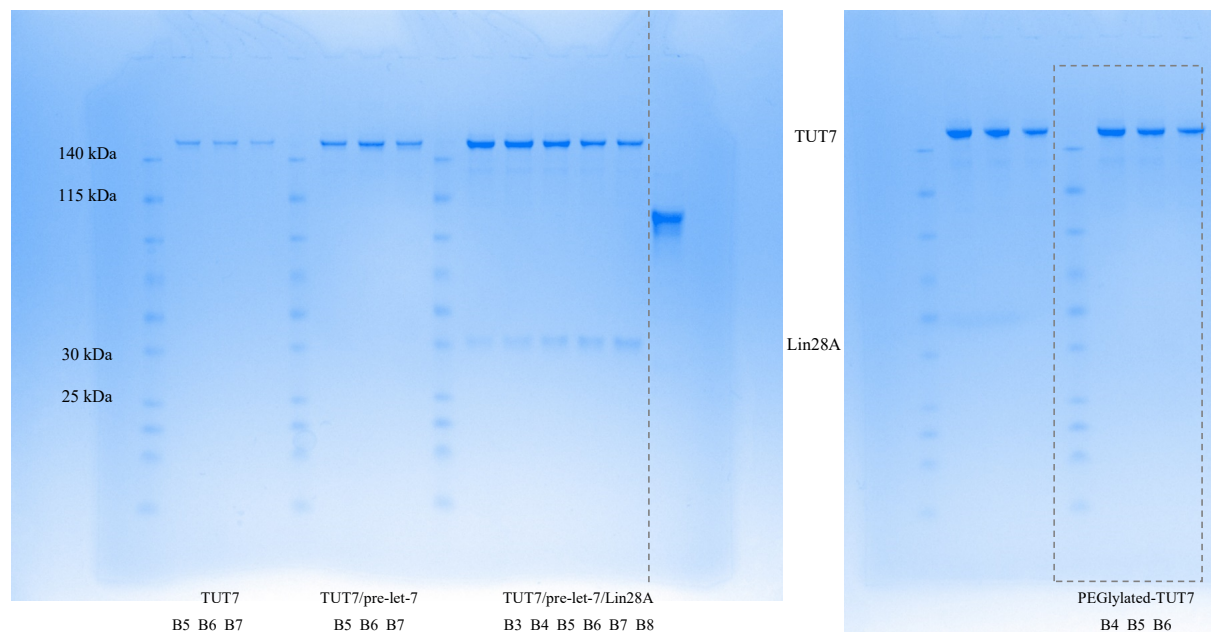

ED figure 1F:

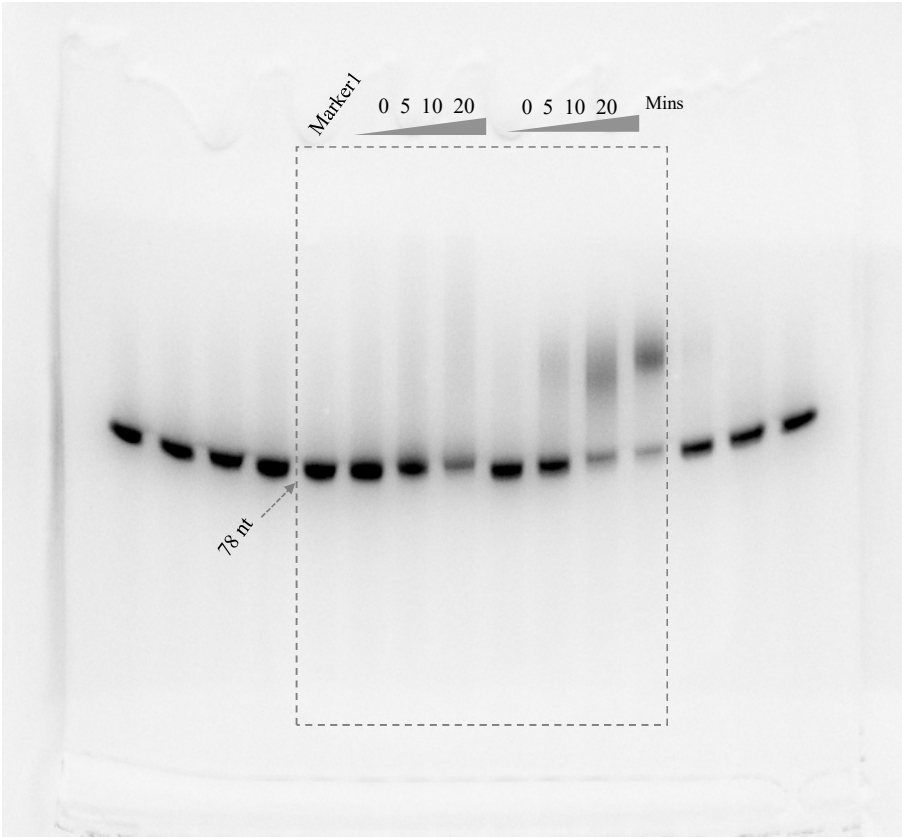

ED figure 1G

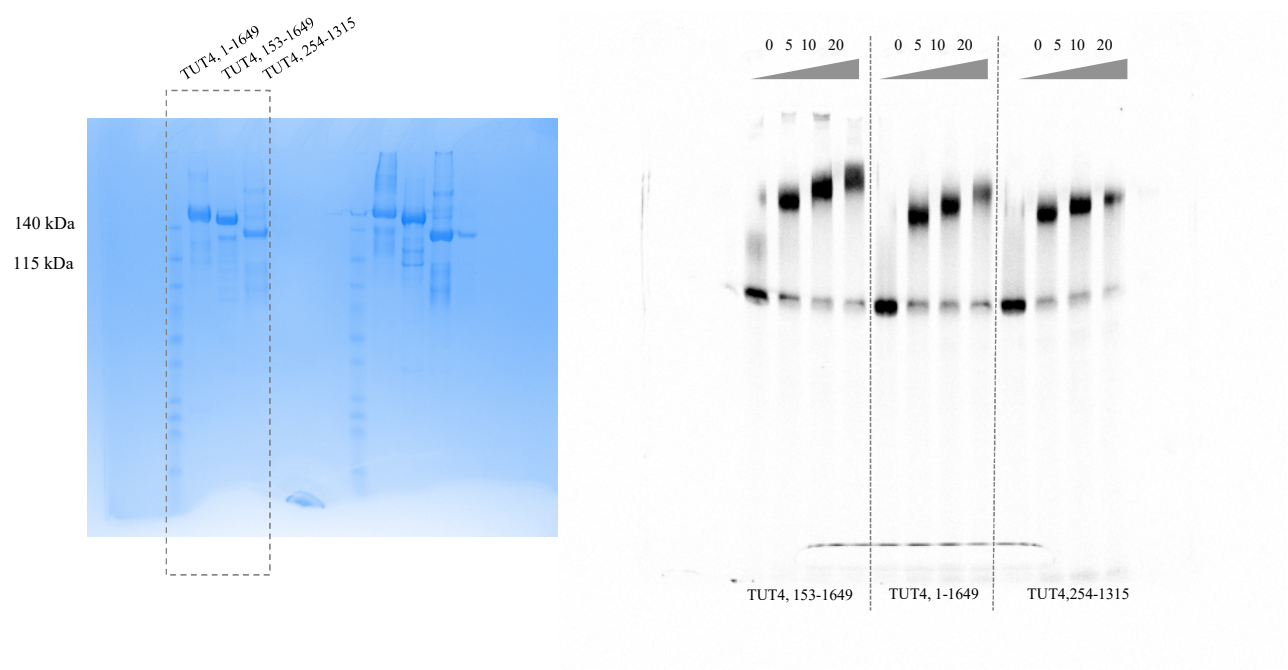

ED figure 1 and J:

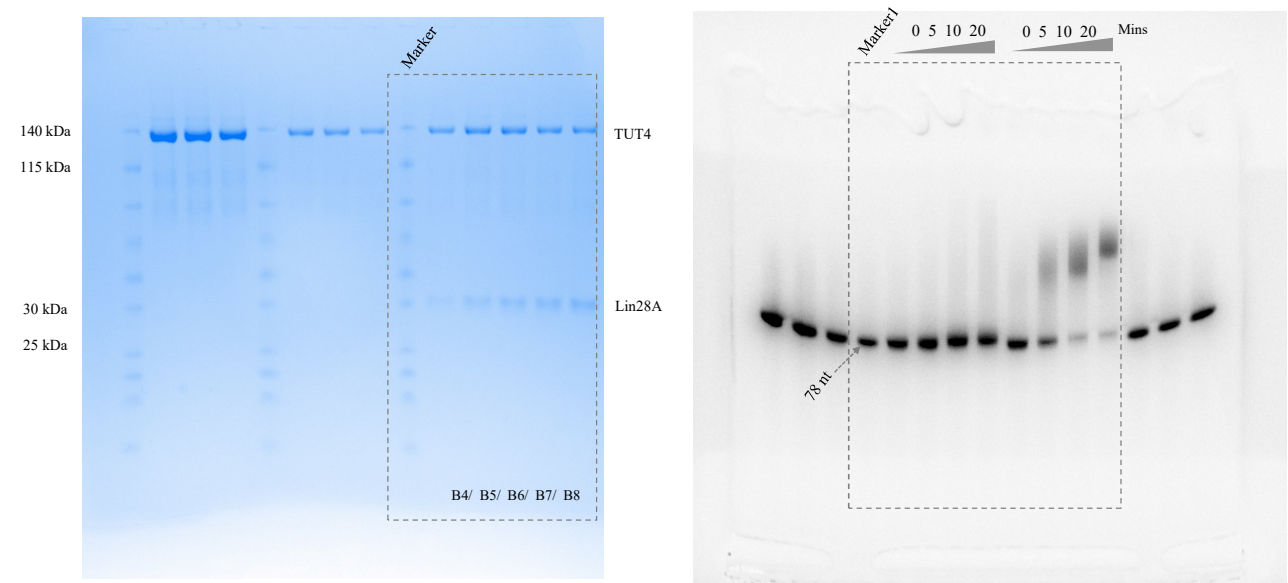

Supplement: Supplementary file 9 — Uncut gel images for Extended Data Fig. 1 panels. [file 41594_2024_1357_MOESM9_ESM.pdf]
